# Diffusion tensor image analysis along the perivascular space (DTI-ALPS): redefining its interpretation and role

**DOI:** 10.1007/s11604-026-02035-0

**Published:** 2026-06-16

**Authors:** Toshiaki Taoka, Rintaro Ito, Rei Nakamichi, Toshiki Nakane, Shinji Naganawa

**Affiliations:** 1 Department of Innovative Biomedical Visualization (iBMV), Nagoya University Graduate School of Medicine, 65 Tsurumai-cho, Showa-ku, Nagoya, Aichi 466-8550 Japan; 2 Department of Radiology, Nagoya University Graduate School of Medicine, Nagoya, Aichi Japan

**Keywords:** DTI-ALPS, Glymphatic system, White matter microstructure, Brain health, MRI

## Abstract

Diffusion tensor image analysis along the perivascular space (DTI-ALPS) was originally developed and defined as a noninvasive diffusion MRI method intended to evaluate glymphatic function and is now widely used in neuroimaging research. However, accumulating evidence suggests that the biological meaning of the ALPS index is more complex than initially assumed as a simple marker of glymphatic function. The ALPS index does not directly measure whole-brain fluid transport but rather reflects localized directional diffusivity based on the Brownian motion of water molecules. Its value is strongly influenced by white matter microstructure, including fiber orientation, crossing fibers, extracellular geometry, and age-related diffusivity changes. In addition, because the ALPS index is a ratio-based measure derived from directional diffusivity components, its alterations may arise from different combinations of numerator and denominator diffusivity changes, indicating that similar ALPS index reductions may reflect distinct underlying microstructural mechanisms. White matter microstructure may represent not merely a confounding factor, but a structural substrate guiding interstitial fluid transport itself. White matter hydraulic permeability exhibits strong anisotropy, and brain fluid transport may occur preferentially along white matter tracts. Thus, the structure dependence of the ALPS index may reflect physiologically relevant interactions between white matter architecture and interstitial fluid dynamics rather than simple measurement bias. In this review, we propose redefining the ALPS index not as a direct marker of glymphatic function, but as a “spatially fixed-point biomarker” evaluating directional diffusivity within anatomically defined regions. Within this framework, the ALPS index can be understood as a composite biomarker reflecting interactions among white matter microstructure, extracellular environment, vascular geometry, and neurofluid-related tissue environment. Because the ALPS index has been associated with aging, sleep, and various diseases, it may function less as a disease-specific marker and more as an adjunctive imaging marker relevant to brain health assessment and the brain tissue environment.

## Introduction

In recent years, the glymphatic system hypothesis, proposed as a mechanism for waste clearance in the brain, has attracted considerable attention, and various imaging biomarkers have been introduced to evaluate brain interstitial fluid dynamics noninvasively [1–4]. However, the definition of “glymphatic function” itself and the extent to which it can be assessed using imaging remain incompletely established [5–9]. Current understanding suggests that brain fluid dynamics represent a complex, multi-pathway phenomenon involving the perivascular space, interstitial space, vascular system, and meningeal lymphatic pathways, among others, and the glymphatic system is often regarded as a conceptual framework integrating these components [2, 3, 10–12]. Such abnormalities in brain fluid dynamics have also been proposed to be collectively understood under the concept of CNS interstitial fluidopathy [13, 14]. Within such a complex framework, interpretation of any single imaging biomarker as a direct measure of glymphatic function has inherent limitations. Within this conceptual framework, brain fluid dynamics are considered part of a broader tissue environment involving interactions among interstitial fluid transport, white matter microstructure, vascular function, and extracellular architecture. From this perspective, imaging biomarkers associated with interstitial fluid dynamics may also be interpreted as markers reflecting overall brain tissue health rather than a single clearance mechanism.

The DTI-ALPS (diffusion tensor image analysis along the perivascular space) method was proposed in 2017 as a diffusion MRI–based approach for evaluating directional diffusivity in relation to the perivascular space [15]. Since then, the method has been widely applied in studies of neurological disorders and aging, and the ALPS index has become broadly interpreted as an imaging biomarker associated with glymphatic function [16–29]. However, accumulating evidence has suggested that the ALPS index is also strongly influenced by white matter microstructure and directional diffusion properties, and does not necessarily correspond to findings from tracer-based studies [5, 30–35]. At the same time, recent studies have suggested that brain fluid transport itself may depend on white matter microstructure and extracellular geometry [2, 9]. These observations indicate that interpretation of the ALPS index requires reconsideration beyond a simple framework of glymphatic imaging.

The aim of this review is to re-examine the interpretation of the DTI-ALPS method and to reconsider its role within neuroimaging research. In particular, we discuss the ALPS index as a structure-dependent diffusion biomarker reflecting interactions between white matter microstructure and interstitial dynamics, and propose its conceptualization as a spatially fixed-point diffusion biomarker applicable to longitudinal and multimodal neurofluid imaging studies. Previous reviews and critical commentaries have emphasized that the ALPS index is influenced by white matter microstructure, tensor orientation, ROI placement, acquisition parameters, and preprocessing procedures, and therefore should not be interpreted as a direct measure of whole-brain glymphatic clearance [31–34]. Building on these observations, the present review aims not simply to reiterate these limitations, but to reorganize the interpretation of DTI-ALPS within a more practical framework. We propose that DTI-ALPS should be understood as a structure-dependent, spatially fixed-point diffusion biomarker that evaluates localized directional diffusivity within anatomically defined periventricular white matter regions, and that it may serve as an adjunctive imaging marker of brain tissue environment and longitudinal brain health-related changes.

## Original concept of DTI-ALPS

**Fig. 1:**
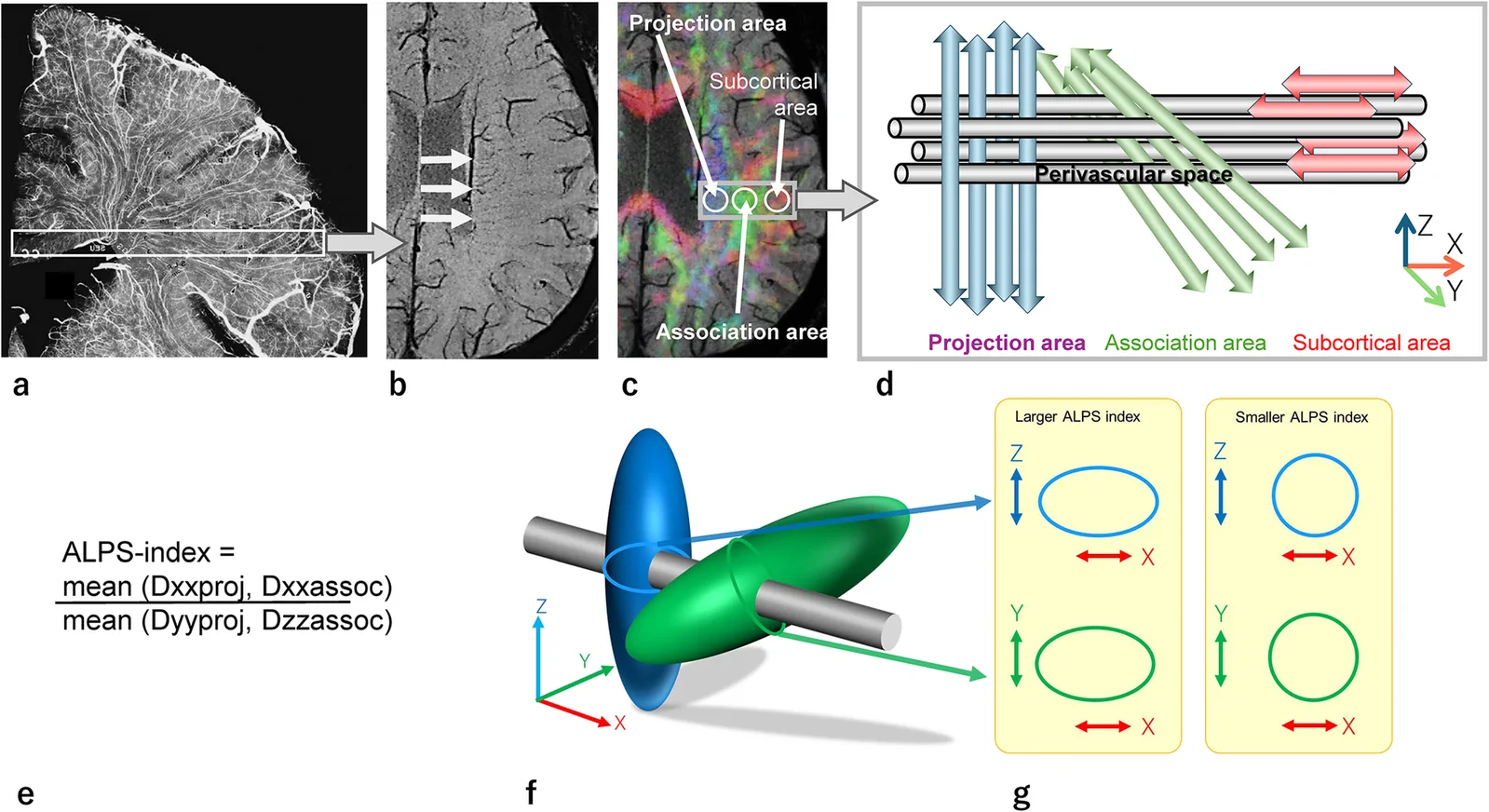
Original concept of the DTI-ALPS method. (**a**) X-ray image of a coronal brain specimen at the level of the lateral ventricular body after contrast injection. Medullary vessels running predominantly in the right–left direction are demonstrated (white box). (**b**) Axial susceptibility-weighted imaging (SWI) at the level of the lateral ventricular body. Medullary vessels and their accompanying perivascular spaces (PVSs) predominantly run in the right–left direction (x-axis). (**c**) DTI color map overlaid on the SWI image. Projection fibers (z-axis direction, blue), association fibers (y-axis direction, green), and subcortical fibers (x-axis direction, red) are shown. Regions of interest (ROIs) are placed within the projection, association, and subcortical areas to measure directional diffusivities along the x-, y-, and z-axes. (**d**) Schematic illustration of the relationship between the perivascular space (gray cylinder) and surrounding white matter fibers. The PVS direction (x-axis) is approximately orthogonal to both projection fibers (z-axis) and association fibers (y-axis). This geometric relationship enables relative evaluation of diffusivity along the PVS direction while reducing the influence of anisotropic diffusion originating from white matter fibers. (**e**) Formula for calculation of the ALPS index. Diffusivities along the x-axis within the projection and association fiber areas (Dxxproj and Dxxassoc) are used as numerator components, whereas diffusivities orthogonal to the PVS direction (Dyyproj and Dzzassoc) are used as denominator components as internal references. (**f**) Schematic illustration of diffusion tensor ellipsoids. The principal axes of the ellipsoids reflect white matter fiber orientation, demonstrating directional dependence in projection fibers (blue) and association fibers (green). (**g**) Conceptual illustration of the ALPS index. Relatively larger x-axis diffusivity components result in higher ALPS index values, whereas relatively smaller x-axis components result in lower values. The original concept of the DTI-ALPS method was to exploit the characteristic orthogonal relationship between the perivascular spaces and major white matter fiber tracts around the lateral ventricular body in order to relatively isolate diffusivity along the PVS direction while minimizing the influence of anisotropic diffusion originating from white matter fibers [15, 33, 36]. This figure illustrates the design concept and geometric assumptions underlying the DTI-ALPS method and serves as the basis for understanding the structure-dependent properties discussed later in this review

The DTI-ALPS method is a diffusion MRI–based analytical approach first proposed in 2017 as a noninvasive method for evaluating glymphatic system–related diffusivity in humans [15]. The basic concept of the method is to exploit the geometric relationship between the perivascular space (PVS) and surrounding white matter fibers at the level of the lateral ventricular body. At this anatomical level, medullary vessels and their accompanying PVS predominantly run in the right–left direction (x-axis on axial images), whereas the major white matter fiber tracts are oriented approximately perpendicular to this direction [36]. This geometric relationship enables relative isolation of diffusion components aligned with the PVS direction while reducing the influence of anisotropic diffusion from white matter fibers (Fig. 1).

To reduce the effects of global diffusivity variation and interindividual differences, the ALPS method was designed as a ratio-based index incorporating an internal reference. Diffusivities along the x-axis within the projection fiber and association fiber regions are used as numerator components, whereas diffusivities measured in directions orthogonal to both the principal fiber orientation and the PVS direction are used as denominator components. Accordingly, higher ALPS index values indicate relatively greater diffusion along the PVS direction and were originally interpreted as reflecting preserved water mobility along the PVS and, by extension, relatively preserved glymphatic function.

**Fig. 2:**
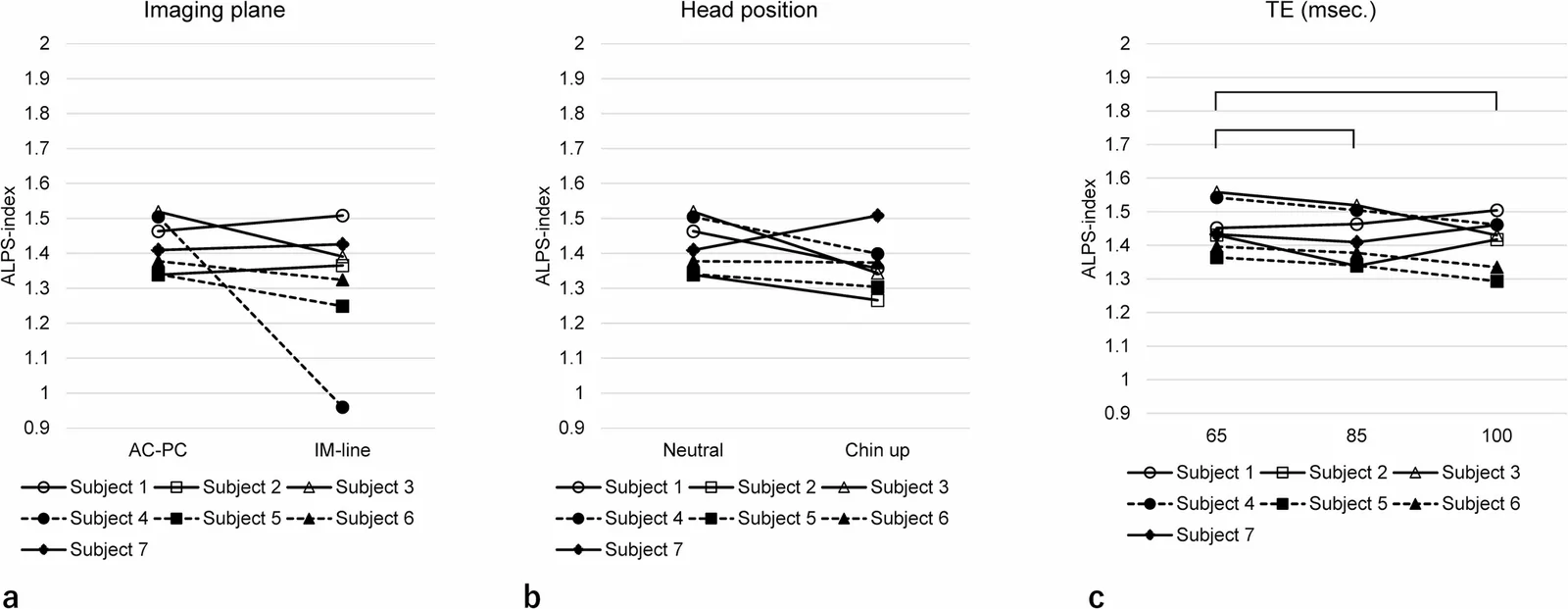
Influence of imaging conditions on the ALPS index. (**a**) Effect of imaging plane. Bilateral ALPS index values calculated from images acquired along the anterior commissure–posterior commissure (AC-PC) line and the infraorbital meatal (IM) line are shown. The intraclass correlation coefficient (ICC) between the two imaging planes was 0.19, indicating poor reproducibility. (**b**) Effect of head position. Bilateral ALPS index values calculated from images acquired in the neutral position and the chin-up position (approximately 20° elevation) are shown. The ICC associated with head position change was 0.22, also indicating poor reproducibility. (**c**) Effect of echo time (TE). Bilateral ALPS index values obtained at TE = 65 ms, 85 ms, and 100 ms are shown. ALPS index values at TE = 65 ms were significantly higher than those at TE = 85 ms and 100 ms (*p* < 0.05). These findings indicate that although the ALPS index demonstrates good reproducibility under fixed acquisition conditions, it can vary depending on geometric and imaging parameters such as imaging plane, head position, and echo time [37]

The ALPS index is a dimensionless ratio typically distributed within a relatively narrow range in human studies, with healthy individuals generally showing values around 1.3 to 1.7 in the original report [15]. Subsequent studies demonstrated that, although the ALPS index shows relatively good reproducibility under standardized conditions, it is influenced by factors such as head position, imaging plane, and acquisition parameters [37] (Fig. 2). In addition, because of the limited spatial resolution of diffusion MRI, the method cannot distinguish periarterial from perivenous spaces or separate diffusion within the PVS from diffusion originating in surrounding tissue compartments.

## Expansion and current interpretations of DTI-ALPS

Since its introduction, the DTI-ALPS method has rapidly expanded in application because of its noninvasiveness, computational simplicity, and compatibility with retrospective analyses using existing datasets. As a result, the method has been investigated in a wide range of conditions, including neurodegenerative diseases, cerebral small vessel disease, idiopathic normal pressure hydrocephalus, traumatic brain injury, demyelinating disorders, sleep-related disorders, and aging [16–18, 23, 24, 28, 38–47]. Across these studies, relatively consistent trends have been reported. In many conditions, lower ALPS index values have been associated with cognitive decline, motor dysfunction, or greater disease severity, leading to broader recognition of the ALPS index as a biomarker associated with various clinical measures.

Interpretation of the ALPS index has been closely linked to glymphatic function since the original report, in which the method was introduced as an approach for evaluating glymphatic function and the reduced ALPS index observed in Alzheimer’s disease was interpreted in relation to impaired clearance mechanisms [15]. This early conceptual positioning likely established a framework in which the ALPS index was understood not only as a marker reflecting the relative predominance of diffusion along the PVS direction, but also as an imaging surrogate of glymphatic function. Consequently, in many subsequent studies, lower ALPS index values have generally been interpreted as suggesting impaired clearance-related function, whereas higher values have been regarded as reflecting relatively preserved function.

## What does DTI-ALPS actually measure?

### Directional diffusivity and Brownian motion

In the DTI-ALPS method, as in diffusion tensor MRI in general, the measured signal is based on the random thermal motion of water molecules (Brownian motion), and the observed diffusion anisotropy is strongly influenced by white matter microstructure and tissue geometry [48–51]. This motion is described as probabilistic displacement and expressed as directional diffusivity along a given orientation. Accordingly, the information obtained from diffusion MRI reflects probabilistic displacement behavior within tissue rather than unidirectional fluid flow. Therefore, increases or decreases in diffusivity along a particular direction should not be interpreted as directly indicating increased or decreased bulk transport in that direction, but rather as reflecting underlying structural characteristics of the tissue environment.

### Deconstructing the ALPS index

Studies focusing on the directional diffusivity components constituting the DTI-ALPS index (numerator and denominator) have demonstrated that changes in the ALPS index do not arise from a single mechanism, but can instead be classified into several distinct patterns. Specifically, reductions in the ALPS index may result from decreased numerator components (x-axis diffusivity), increased denominator components (y- and z-axis diffusivities), or a combination of both.

In some conditions, reductions in the ALPS index are primarily explained by decreases in the numerator components. For example, studies of late-onset epilepsy and repetitive transcranial magnetic stimulation (rTMS) in patients with insomnia demonstrated reduced x-axis diffusivity in the projection fiber area (Dxxproj), whereas the denominator components showed no clear changes [52, 53]. Similar patterns have also been reported in type 2 diabetes mellitus and acute lymphoblastic leukemia [54, 55]. These findings correspond to a so-called “numerator-driven pattern.”

In contrast, many studies have suggested that changes in the ALPS index are primarily driven by increases in the denominator components. In migraine, mild cognitive impairment (MCI), diffuse axonal injury (DAI), and chronic tinnitus, elevated y- and z-axis diffusivities contributed to reduced ALPS index values despite the absence of clear changes in the numerator components [40, 56–58]. This tendency appears even more pronounced in idiopathic normal pressure hydrocephalus (iNPH), in which x-axis diffusivity is preserved or even increased, whereas relative increases in the denominator components are thought to contribute to ALPS index reduction [16, 38, 59]. Similar tendencies have also been reported in Parkinson’s disease, juvenile myoclonic epilepsy, and obstructive sleep apnea, where ALPS index reduction was predominantly associated with increased denominator diffusivities [24, 39, 60, 61].

In addition, a large-scale population study by Satpathi et al. suggested that the ALPS index cannot be explained solely by perivascular diffusion, but is influenced by multiple directional diffusivity components [5]. Consistent with this, another recent population-based study demonstrated that reductions in the ALPS index were predominantly associated with increases in denominator components, suggesting that age- and systemic condition–related white matter diffusivity changes may substantially influence ALPS index [62]. These findings can also be regarded as representative of a “denominator-driven pattern.”

However, findings in some conditions cannot be readily attributed to alterations in a single component of the ALPS index. In studies of Parkinson’s disease–related cognitive impairment, directional diffusivity analysis demonstrated that changes in y-axis diffusivity within the projection fiber area and z-axis diffusivity within the association fiber area were associated with cognitive decline and progression to dementia [60]. Similarly, in cluster headache, although reduced ALPS index values have been reported, the pattern of directional diffusivity changes was not clearly defined, making it difficult to classify the findings into a simple numerator- or denominator-driven pattern [63]. Furthermore, in cancer-related pain, reductions in diffusivity were observed in both numerator and denominator components [64], suggesting that ALPS index alterations cannot always be explained by changes in a single component alone.

These findings indicate that even when similar reductions in the ALPS index are observed, the underlying patterns of directional diffusivity changes are not necessarily the same. In other words, a single ratio such as the ALPS index has inherent limitations in uniquely identifying the underlying physiological or microstructural alterations. Taken together, these observations suggest that the ALPS index should not be interpreted as a direct measure of a single physiological process, particularly brain clearance function, but rather as a composite diffusion biomarker influenced by directional diffusivity and tissue microstructure. To facilitate practical interpretation, Table 1 summarizes a conceptual framework for ALPS index changes based on the relative contributions of numerator and denominator diffusivity components (Table 1).

**Table 1:** Conceptual framework for interpreting ALPS index changes based on numerator and denominator diffusivity components

Pattern	Component-level change	Effect on ALPS index	Interpretive consideration
Numerator-driven reduction	Decreased numerator diffusivity components with relatively preserved denominator components	Decreased	May indicate reduced x-axis directional diffusivity within the numerator regions. However, this pattern should not be interpreted automatically as reduced glymphatic clearance, because numerator components may also be influenced by local tissue structure, vascular/perivascular geometry, and acquisition-related factors.
Denominator-driven reduction	Increased denominator diffusivity components with relatively preserved numerator components	Decreased	Indicates that ALPS index reduction may occur even without reduced numerator diffusivity. This pattern may reflect changes in white matter microstructure, extracellular geometry, tissue organization, or non-PVS directional diffusivity components.
Mixed or complex reduction	Concurrent or variable changes in both numerator and denominator components	Decreased or variable	Suggests heterogeneous or combined mechanisms. Interpretation requires examination of individual directional diffusivity components rather than relying on the ALPS ratio alone.
ALPS index increase	Increased numerator components and/or decreased denominator components	Increased	May reflect relatively greater numerator diffusivity, lower denominator diffusivity, or both. This pattern should not be interpreted automatically as enhanced glymphatic function, because it may also reflect white matter structural properties, extracellular geometry, or technical factors.

### Influence of white matter microstructure

Directional diffusivities measured by diffusion MRI can be regarded as indicators reflecting the underlying tissue structures that constrain water motion. Accordingly, diffusion anisotropy in white matter is determined not by a single factor, but by multiple microstructural features, including fiber orientation, crossing fibers, orientation dispersion, and axonal morphology [65–67].

In the DTI-ALPS method, the diffusivities used in the denominator are assumed to function as neutral internal references unaffected by the perivascular space. However, this assumption is not supported by the known properties of white matter diffusion. Traditionally, radial diffusivity in white matter has often been assumed to be symmetric, with the second and third eigenvalues (λ2 and λ3) considered nearly equivalent. In practice, however, λ2 ≠ λ3 is widely observed [34, 63, 64]. The mechanisms underlying this asymmetry are likely heterogeneous and may reflect multiple structural factors, including crossing fibers, orientation dispersion, and axonal undulation. Although these effects cannot be attributed to a single mechanism, the assumption of symmetric diffusion in white matter is clearly not universally valid.

**Fig. 3:**
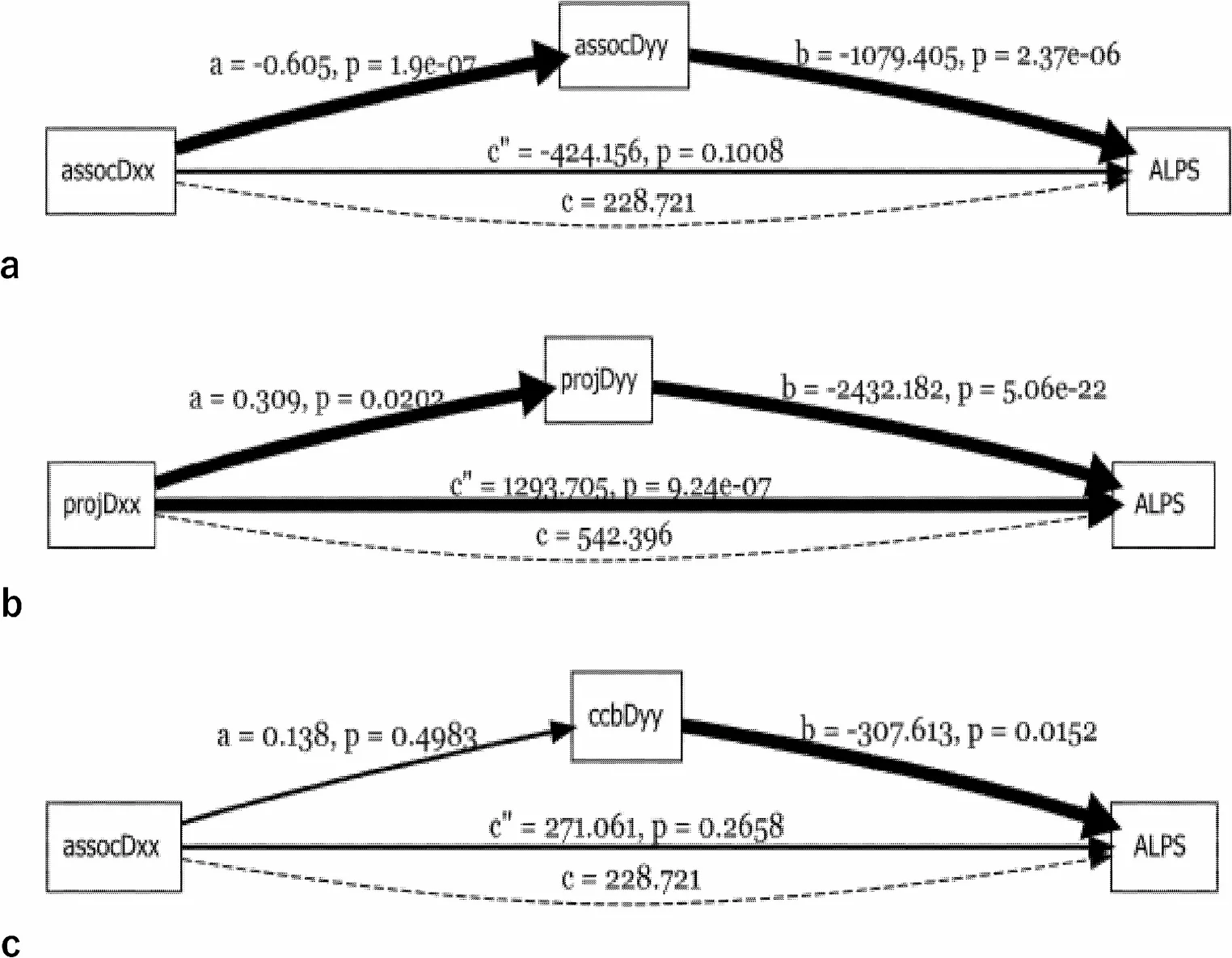
Mediation analysis demonstrating the contribution of white matter diffusivity to the ALPS index. (**a**) Mediation effect of y-axis diffusivity within the association fiber area (assocDyy) on the relationship between x-axis diffusivity in the association fiber area (assocDxx) and the ALPS index. A significant mediation pathway through assocDyy was observed, suggesting that the ALPS index is influenced not only by diffusivity along the perivascular space (PVS) direction, but also by non-PVS directional diffusivity within the association fiber region. (**b**) Mediation effect of y-axis diffusivity within the projection fiber area (projDyy) on the relationship between x-axis diffusivity in the projection fiber area (projDxx) and the ALPS index. A similarly significant mediation effect was observed in the projection fiber region, further supporting the concept that the ALPS index is a composite metric dependent on intra–white matter diffusivity properties. (**c**) Mediation effect of y-axis diffusivity within the corpus callosum body (ccbDyy). Commissural fiber diffusivity outside the original ALPS ROI was also associated with the ALPS index, suggesting that the ALPS index is influenced not only by local PVS-related diffusivity, but also by broader white matter microstructural properties. These findings indicate that the ALPS index should not be interpreted as a simple perivascular diffusion marker, but rather as a structure-dependent composite diffusion index reflecting both white matter microstructure and PVS-related diffusion components [68]

Furthermore, these structure-dependent effects are not merely theoretical considerations, but have also been demonstrated in empirical data. In our study, the ALPS index showed significant associations with diffusion properties within white matter, particularly with white matter structures such as commissural fibers. In addition, mediation analysis identified pathways through which intra–white matter diffusivity contributed to the ALPS index, suggesting that the ALPS index does not solely reflect diffusion along the perivascular space [68] (Fig. 3).

Taken together, the ALPS index is strongly influenced by white matter microstructure while also partially reflecting diffusion components related to vascular and perivascular structures. Therefore, it should not be regarded as representing a single physiological function, but rather as a composite diffusion index integrating both white matter structural effects and perivascular-related diffusion components.

## Relationship to intrathecal GBCA study

In recent years, comparative studies between the DTI-ALPS method and intrathecal GBCA studies have increasingly examined the relationship between the ALPS index and intrathecal tracer dynamics. In particular, Mossige et al. reported that the ALPS index showed only limited correspondence with cortical tracer dynamics and demonstrated a negative association with deep white matter tracer retention, leading the authors to question whether DTI-ALPS adequately reflects glymphatic function [35].

Indeed, there are inherent limitations in interpreting the ALPS index as a direct measure of whole-brain glymphatic function. The DTI-ALPS method evaluates directional diffusivity within a limited white matter region adjacent to the lateral ventricular body and does not directly visualize global CSF–ISF exchange or metabolic waste clearance. Moreover, as discussed above, DTI-ALPS reflects localized diffusion properties derived from Brownian motion and may therefore characterize a different spatial and physiological scale of interstitial fluid dynamics from the macroscopic bulk transport observed in tracer studies [8, 9, 48, 50].

However, such discrepancies should not immediately be regarded as evidence against the physiological relevance of the ALPS index. Rather, these methods may evaluate interstitial fluid dynamics at different spatial scales and physiological hierarchies. Whereas intrathecal tracer MRI assesses relatively macroscopic CSF redistribution and bulk transport across the brain, DTI-ALPS may reflect localized microscopic directional diffusivity within deep white matter, namely microscopic interstitial water mobility.

Even regarding the negative association between the ALPS index and deep white matter tracer retention discussed by Mossige et al., caution is warranted before attributing the finding solely to ventricular reflux. In a study by Zhang et al. involving patients with cerebral small vessel disease, a similar negative association between the ALPS index and tracer retention was observed despite the absence of ventricular reflux as a primary pathological feature [69]. These findings suggest that the ALPS index may reflect aspects of interstitial fluid dynamics abnormalities shared within deep white matter, rather than merely representing ventricular reflux phenomena specific to idiopathic normal pressure hydrocephalus.

Current understanding increasingly recognizes brain interstitial fluid dynamics as a complex, multi-pathway phenomenon involving the perivascular space, interstitial space, vascular system, and meningeal lymphatic pathways [1, 9, 11]. Accordingly, there is no universally accepted definition of glymphatic function with respect to either spatial scale or physiological hierarchy. At the same time, the limited correspondence between DTI-ALPS and intrathecal tracer MRI remains an important limitation when the ALPS index is interpreted as a glymphatic biomarker. Discordant findings should not be minimized, because they indicate that the ALPS index cannot be assumed to directly represent tracer-based CSF–ISF exchange or whole-brain glymphatic transport. Therefore, DTI-ALPS should be interpreted as reflecting localized directional diffusivity within a specific white matter region rather than direct tracer movement. From this perspective, DTI-ALPS and intrathecal tracer MRI should not be regarded as mutually exclusive measures, but rather as complementary modalities reflecting different aspects of interstitial fluid dynamics.

## A Framework for Interpreting DTI-ALPS

The ALPS index is a metric based on directional diffusivity within a geometrically constrained brain region, and its value is influenced by multiple factors, including interstitial fluid dynamics, white matter microstructure, extracellular geometry, and the vascular environment. Although these characteristics are not specific to a single glymphatic clearance mechanism, they may nonetheless be intrinsically related to the structural and physiological state of brain tissue. Accordingly, it is not appropriate to interpret DTI-ALPS within a simple binary framework of whether it is or is not a pure glymphatic marker. Rather, the ALPS index should be understood as a marker reflecting localized directional diffusivity associated with brain interstitial fluid dynamics. From this perspective, DTI-ALPS may be reconsidered not as a surrogate of a single mechanism, but as a composite biomarker reflecting the brain tissue environment [5, 9, 68].

Furthermore, recent evidence suggests that white matter microstructure itself may serve as a structural substrate guiding brain fluid transport [2, 70]. From this viewpoint, the structure dependence observed in DTI-ALPS may not merely represent a confounding factor, but may partially reflect physiological interactions between white matter architecture and interstitial fluid dynamics. The following section further reconsiders the role of white matter structure in DTI-ALPS from this perspective.

## Rethinking the role of DTI-ALPS

### Structural dependence and composite nature of the ALPS index

Based on these considerations, the perspective on the ALPS index should shift from “what does it measure?” (interpretation) to “how should it be used?” (role). Rather than focusing solely on whether the ALPS index directly reflects glymphatic function, greater importance should be placed on how this metric should be positioned and utilized within neuroimaging.

Directional diffusivities measured by diffusion MRI reflect not water motion itself, but the tissue structures that constrain it. In white matter, directional diffusion is influenced by multiple microstructural factors, including fiber orientation, crossing fibers, orientation dispersion, and axonal morphology. In the DTI-ALPS method, the denominator components are treated as internal references unaffected by the perivascular space; however, white matter diffusion is not necessarily symmetric, and asymmetry between the second and third eigenvalues (λ2 and λ3) has been widely reported [34, 63, 64]. These findings indicate that the ALPS index cannot be explained solely by diffusion along the perivascular space.

**Fig. 4:**
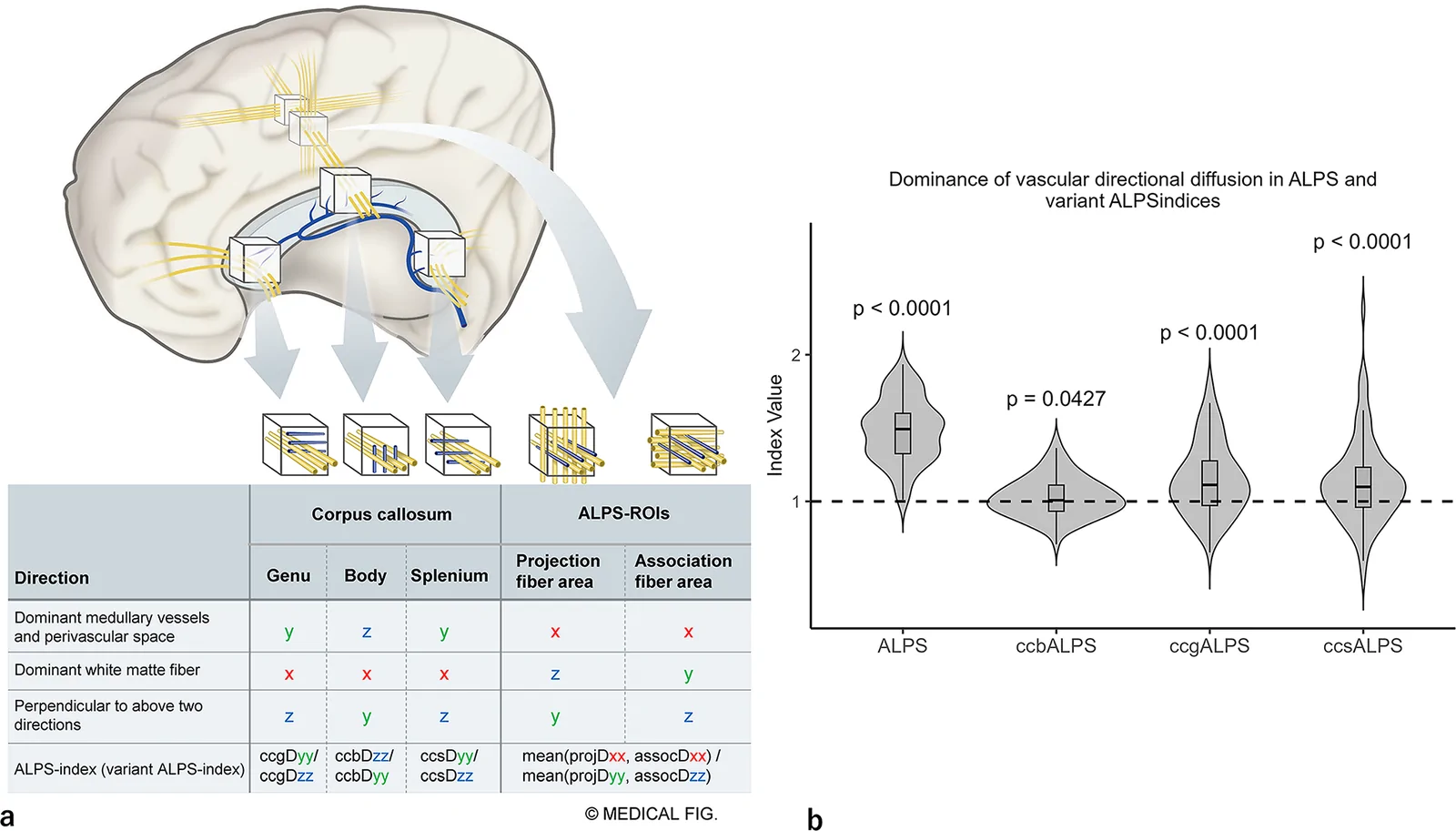
Variant ALPS index analysis in the corpus callosum and residual contribution of perivascular diffusivity. (**a**) Schematic illustration of directional relationships in the corpus callosum and the conventional ALPS ROI. In the original DTI-ALPS method, the ALPS index is calculated by exploiting the orthogonal relationship between the perivascular space (PVS) direction (x-axis) and the principal white matter fiber direction (z-axis in the projection fiber area and y-axis in the association fiber area). In the present analysis, variant ALPS indices were defined within the corpus callosum (genu, body, and splenium), where white matter architecture is relatively simple and crossing fibers are limited. Because the principal fiber orientation in the corpus callosum runs relatively uniformly along the right–left direction (x-axis), the variant ALPS index would theoretically be expected to approach 1 in the absence of diffusivity components related to the PVS direction. (**b**) Violin plots showing distributions of the conventional ALPS index and corpus callosum variant ALPS indices (ccbALPS, ccgALPS, and ccsALPS). In addition to the conventional ALPS index, variant ALPS indices in the genu (ccgALPS) and body (ccbALPS) of the corpus callosum also showed values significantly greater than 1. These findings suggest that diffusivity components related to the PVS direction contribute to the ALPS signal even under conditions in which the influence of white matter microstructure is relatively simplified [68]

As discussed above, our study demonstrated significant associations between the ALPS index and intra–white matter diffusion properties, particularly those related to commissural fibers, and mediation analysis further identified pathways through which white matter diffusivity contributed to the ALPS index [68]. At the same time, the contribution of the perivascular space cannot be completely disregarded. Using a variant ALPS index in the corpus callosum, where white matter architecture is relatively simple, we observed values significantly greater than 1 even under conditions in which the index would theoretically be expected to approach 1 [68] (Fig. 4). This finding suggests that perivascular-related diffusion components also contribute, at least partially, to the measured ALPS index.

Taken together, the ALPS index should be understood as a composite diffusion index strongly influenced by white matter microstructure while also partially reflecting perivascular-related diffusion components.

### White matter microstructure and interstitial fluid dynamics

Importantly, such white matter dependence may not merely represent a “confounding factor,” but rather a structural substrate underlying interstitial fluid dynamics itself. Recent reviews of the glymphatic system have described white matter tracts as “privileged pathways” for interstitial transport, suggesting that brain fluid transport may occur preferentially along white matter fiber orientations [2, 70, 71].

This concept is supported by studies examining the relationship between white matter microstructure and interstitial transport. Hydraulic permeability in white matter has been shown to be greater parallel to fiber orientation and lower in the perpendicular direction [70], indicating that brain fluid transport may be structurally guided by white matter microarchitecture. Rasmussen et al. further emphasized that brain fluid transport should be understood not as simple diffusion, but as a dynamic system involving periarterial influx, extracellular transport, and perivenous efflux [9]. In addition, studies of convection-enhanced delivery (CED) have demonstrated that the distribution of tracers and therapeutic agents occurs preferentially along white matter fiber pathways and is strongly influenced by local microstructure [71, 72].

These findings suggest that white matter microstructure may actively shape interstitial fluid dynamics rather than merely acting as a background confounder. From this perspective, the structure dependence observed in DTI-ALPS may reflect physiologically relevant information linked to interstitial fluid dynamics rather than simple measurement bias. Accordingly, the ALPS index may be better regarded not as a direct functional marker of the glymphatic system, but as a structure-dependent biomarker reflecting interactions between white matter architecture and interstitial fluid dynamics.

**Fig. 5:**
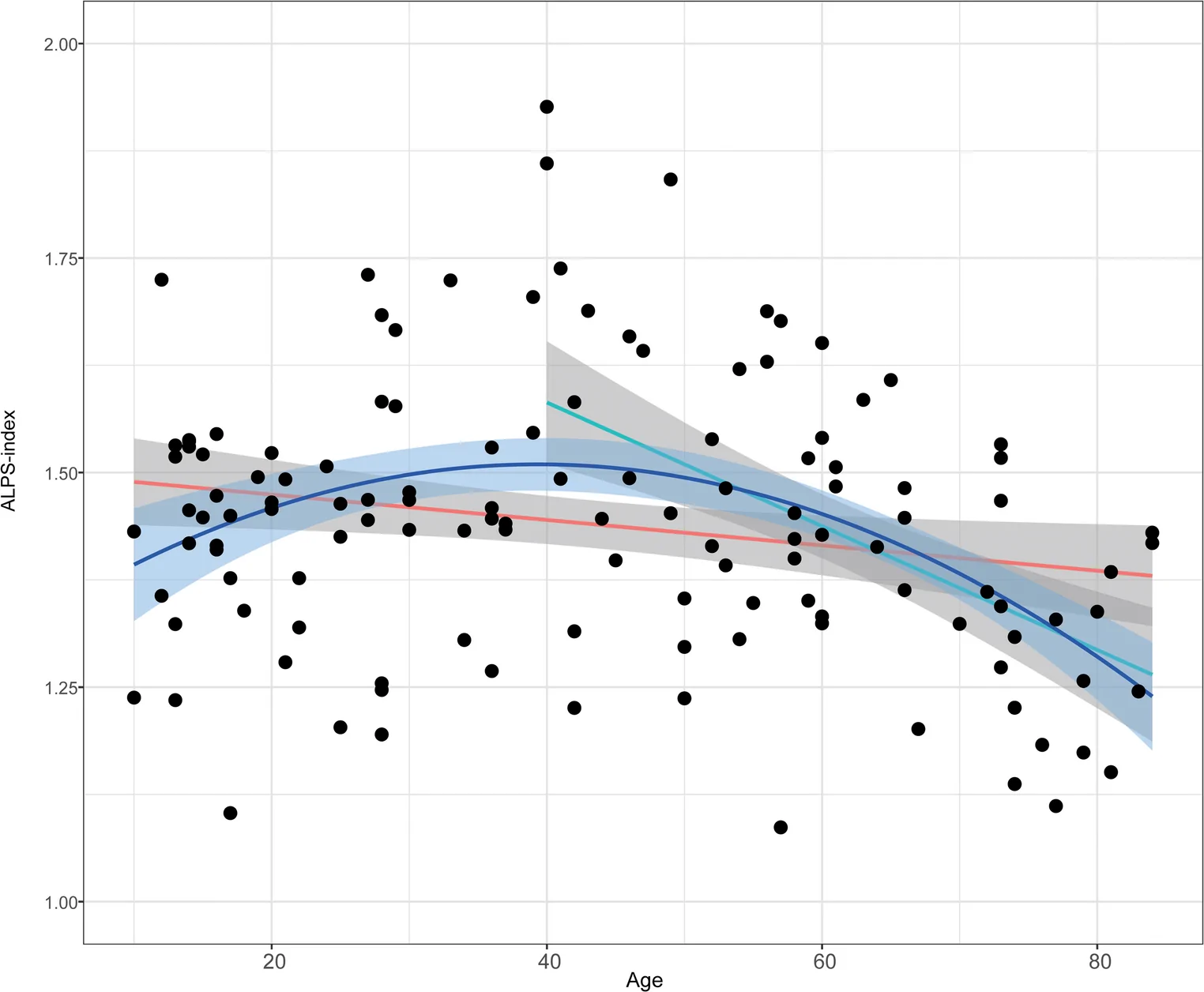
Age dependency of the ALPS index in normal aging. Scatter plot showing the relationship between the ALPS index and age in healthy subjects. Black dots represent individual ALPS index values. The red line indicates linear regression for all subjects, the light blue line indicates linear regression for subjects older than 40 years, and the blue line represents the quadratic regression curve. Gray and blue shaded areas indicate the corresponding 95% confidence intervals. The ALPS index demonstrated a nonlinear trajectory with a peak around middle age rather than a simple linear decline, suggesting that age-related ALPS index alterations cannot be explained as a unidirectional process alone [18]. These findings support the concept that the ALPS index reflects a composite tissue environment influenced by multiple factors, including white matter microstructure, interstitial environment, and blood–brain barrier function, rather than simply representing reduced glymphatic function alone

**Fig. 6:**
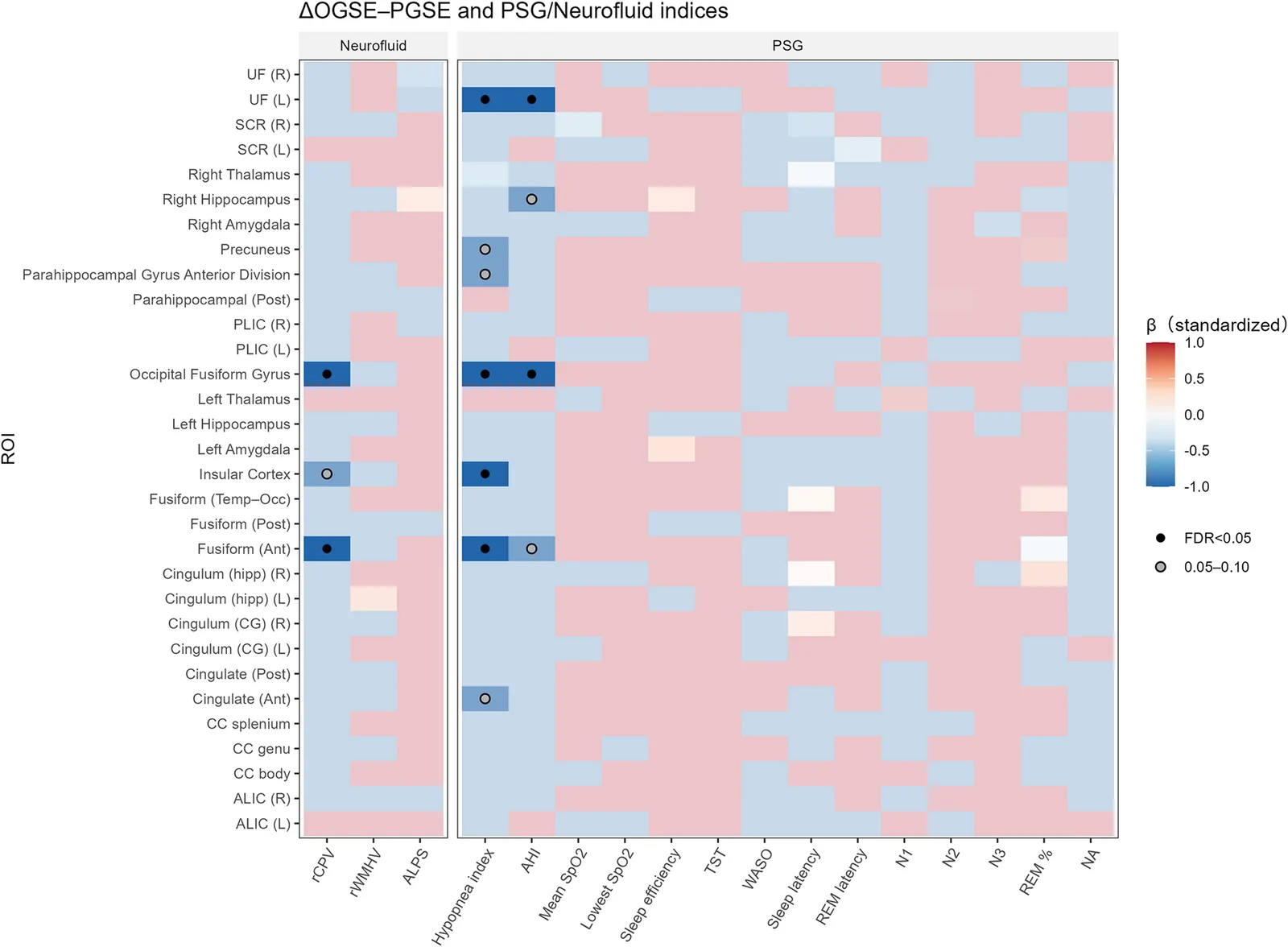
Associations between time-dependent diffusion MRI (ΔOGSE–PGSE) and sleep- and neurofluid-related indices. Heatmap showing ROI-based associations between the time-dependent diffusion MRI metric ΔOGSE–PGSE and polysomnography (PSG) parameters as well as neurofluid-related imaging indices in patients with obstructive sleep apnea (OSA). Colors indicate standardized regression coefficients (β values), with red representing positive associations and blue representing negative associations. Black circles indicate statistical significance after false discovery rate (FDR) correction (FDR < 0.05), whereas white circles indicate trend-level significance (0.05–0.10). ΔOGSE–PGSE demonstrated associations with multiple PSG parameters, including the apnea–hypopnea index (AHI), hypopnea index, mean SpO2, sleep efficiency, and sleep stages across several white matter and subcortical regions. In contrast, associations with neurofluid-related imaging indices, including the ALPS index, relative choroid plexus volume (rCPV), and relative white matter hyperintensity volume (rWMHV), were relatively limited. These findings suggest that white matter microstructure itself may represent an important structural substrate underlying sleep-related brain fluid dynamics [73]

Indeed, in our study of normal aging, the ALPS index demonstrated a nonlinear age-related trajectory with a peak in middle age rather than a simple linear decline [18] (Fig. 5). This finding suggests that age-related ALPS alterations may reflect complex interactions among white matter microstructure, extracellular environment, vascular factors, and interstitial fluid dynamics, rather than a unidirectional reduction in glymphatic clearance alone. In the MOONLIGHT study of obstructive sleep apnea (OSA), the ALPS index was associated with multiple sleep-related parameters, and mediation analysis demonstrated pathways through which intra–white matter diffusivity contributed to the ALPS index [68] (Fig. 3). Furthermore, time-dependent diffusion MRI in the same cohort demonstrated associations between ΔOGSE–PGSE and sleep-disordered breathing indices, including the apnea–hypopnea index (AHI), across multiple white matter and subcortical regions [73] (Fig. 6). These findings support the view that the ALPS index reflects a broader brain tissue environment involving white matter microstructure, neurofluid-related environment, and vascular function rather than a single clearance mechanism. Recent large-scale studies have further demonstrated associations between the ALPS index and aging, cognition, systemic condition, and modifiable vascular factors such as blood pressure [62]. These observations further support the concept that ALPS index reflects integrated properties of the brain tissue environment rather than a single physiological process.

From this perspective, DTI-ALPS is better regarded not as a method for directly visualizing whole-brain transport, but as a “fixed-point diffusion biomarker” evaluating directional diffusivity within a geometrically defined region. The next section revisits the role of DTI-ALPS within this framework.

## DTI-ALPS as a spatially fixed-point diffusion biomarker

In the context of DTI-ALPS, a “spatially fixed-point biomarker” refers to a measurement concept in which directional diffusivity is compared across subjects and over time within a specific brain region where geometric conditions are relatively stable. The ALPS index was designed to evaluate directional diffusivity within a limited region adjacent to the lateral ventricular body under standardized geometric conditions. Although such a fixed-point approach restricts the measurement area, it offers advantages in anatomical reproducibility and measurement consistency.

These characteristics make the ALPS index suitable not only for cross-sectional comparisons between subjects, but also for longitudinal assessment within the same individual [23, 74]. In addition, because the DTI-ALPS method is noninvasive and can be applied retrospectively to existing diffusion MRI datasets, it has practical advantages for large-scale population studies and longitudinal investigations [75, 76].

At the same time, DTI-ALPS remains sensitive to geometric factors such as head position, imaging plane, and tensor orientation. The CHAMONIX study demonstrated that the ALPS index changes in the chin-up position [37], and tensor reorientation techniques have been reported to improve intraclass correlation coefficients (ICCs) [77]. These findings indicate that standardization of both image acquisition and computational procedures is essential for ensuring reproducibility of the ALPS index.

Laterality is another relevant factor for the standardization and interpretation of ALPS index measurements. Although the original DTI-ALPS method was defined using the left hemisphere [15], subsequent studies have used unilateral measurements, bilateral averaging, or separate left–right comparisons. Because no universally established standard regarding laterality is currently available, bilateral averaging may be useful for improving measurement stability in studies of overall brain tissue environment or longitudinal changes, whereas hemisphere-specific ALPS indices may be informative in studies of lateralized pathology or hemispheric asymmetry. Therefore, the laterality of ALPS measurements should be selected according to the study purpose and clearly reported.

Recent efforts toward standardization have included template-based ROI placement methods, automated analysis pipelines, and multicenter harmonization approaches such as ComBat [78–80]. Such developments may further improve the reproducibility, scalability, and multicenter applicability of DTI-ALPS.

Importantly, anatomically localized biomarkers are widely used in clinical medicine as surrogate indicators of broader systemic or organ-level conditions. For example, carotid intima–media thickness and retinal imaging are used to assess vascular and microvascular health beyond the measurement site itself [81–83]. From this perspective, the periventricular white matter evaluated by DTI-ALPS may similarly function as a structurally informative region reflecting broader alterations in brain tissue environment and neurofluid-related dynamics.

This “fixed-point” characteristic is a fundamental feature that makes the ALPS index valuable not only for single time-point disease assessment, but also for longitudinal studies and evaluation of brain health.

## ALPS index as an adjunctive marker of brain health

In this review, “brain health” refers to the integrated state of brain tissue encompassing white matter microstructure, neurofluid-related environment, vascular integrity, metabolic status, and aging-related changes. Although the ALPS index has been associated with a wide range of pathological and systemic conditions, it shows limited disease specificity. Traditionally, this characteristic has been regarded as a limitation; however, it may instead indicate that the ALPS index is broadly sensitive to the brain tissue environment. Indeed, ALPS index changes have been associated with aging, sleep, systemic conditions, and longitudinal changes, suggesting that the ALPS index may reflect the broader brain tissue environment rather than a disease-specific process.

This interpretation is supported by studies examining blood biomarkers. The ALPS index has been reported to correlate with lipid metabolism, glucose metabolism, renal function, and glial fibrillary acidic protein (GFAP), an astrocytic injury marker [84]. These findings suggest that the ALPS index may capture aspects of broader systemic and metabolic conditions related to the brain environment through local diffusion characteristics within the brain.

In addition, the ALPS index may reflect the overall baseline condition of the brain rather than specific focal lesions themselves. In patients with glioblastoma, the ALPS index was not significantly associated with tumor volume or edema, and laterality effects were inconsistent [85]. Furthermore, in glaucoma, reduced ALPS index values have been associated with enlarged perivascular spaces, thinning of visual-related cortical regions, and decreased functional connectivity, and may also mediate relationships with retinal nerve fiber layer thickness [86]. These findings further support the view that the ALPS index may reflect global network integrity and neurofluid-related environment rather than a single focal lesion.

**Fig. 7:**
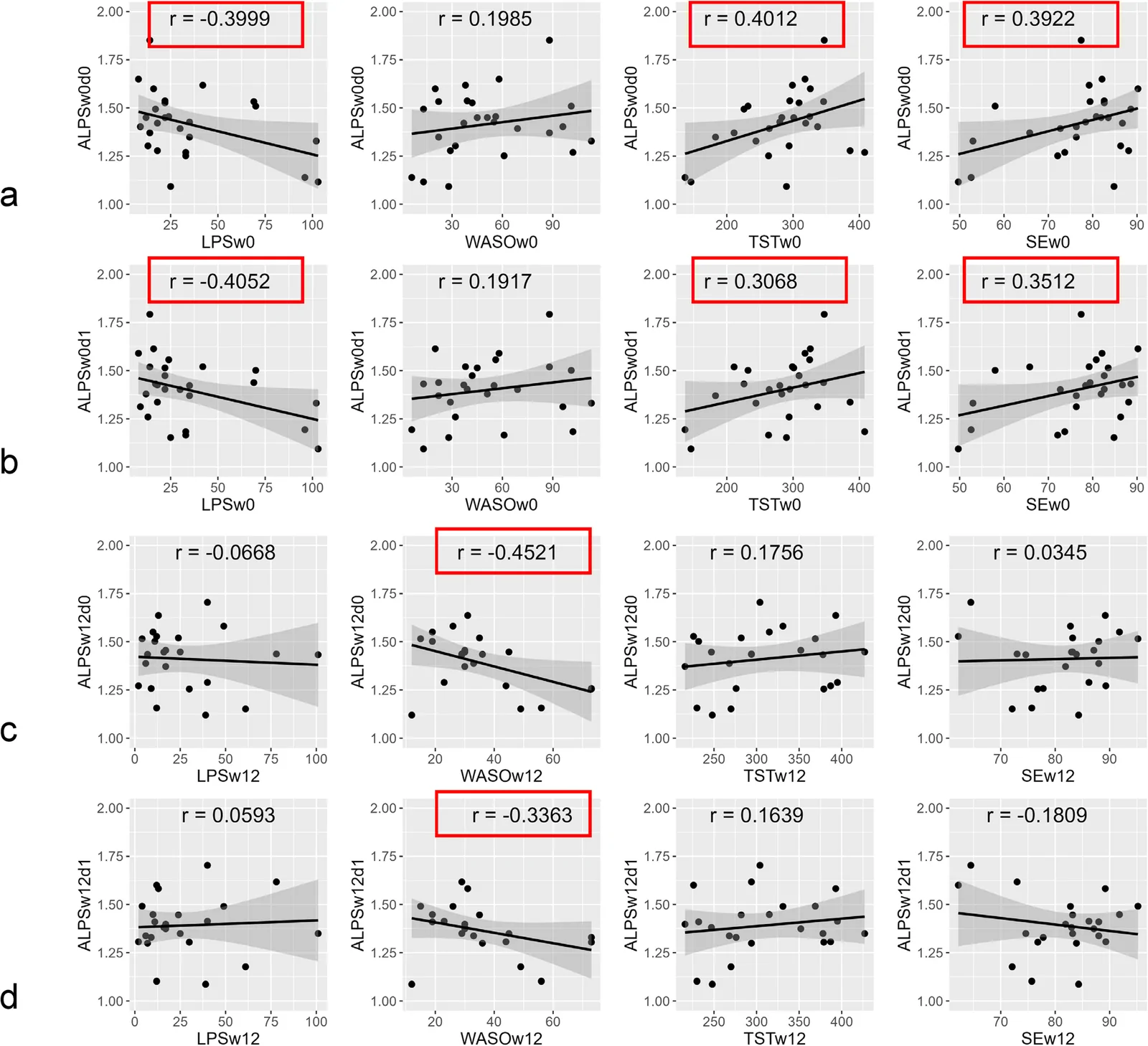
Associations between sleep parameters and the ALPS index in the FLUID study. (**a, b**) Correlations between the ALPS index and objective sleep parameters before lemborexant administration (Week 0: W0). ALPSw0d0 (**a**; evening MRI before overnight sleep examination) and ALPSw0d1 (b; morning MRI after sleep examination) showed negative correlations with latency to persistent sleep (LPS) and positive correlations with total sleep time (TST) and sleep efficiency (SE). No clear association was observed with wake after sleep onset (WASO). (**c, d**) Associations between the ALPS index and sleep parameters after 12 weeks of lemborexant administration (Week 12: W12). ALPSw12d0 (**c**) demonstrated a significant negative correlation with WASOw12, whereas no clear associations with other sleep parameters were observed. ALPSw12d1 (**d**) showed no significant correlations [88]. These findings suggest that the ALPS index may be associated with sleep condition, particularly sleep initiation and sleep maintenance. Red boxes indicate statistically significant associations. LPS = latency to persistent sleep; WASO = wake after sleep onset; TST = total sleep time; SE = sleep efficiency; W0 = baseline before lemborexant administration; W12 = 12 weeks after lemborexant administration; d0 = evening MRI before sleep examination; d1 = morning MRI after overnight sleep examination

Sleep is also an important physiological factor associated with the ALPS index. In a longitudinal study evaluating changes before and after administration of lemborexant, an orexin receptor antagonist, we assessed the ALPS index, blood–brain barrier permeability (Ktrans), and choroid plexus volume (CPV), and examined their relationships with sleep status [87, 88]. In particular, changes in sleep condition were associated with longitudinal changes in the ALPS index, suggesting that the ALPS index may be sensitive to alterations in the sleep-related brain environment (Fig. 7).

Furthermore, the ALPS index may function not only as a static marker, but also as a dynamic biomarker responsive to temporal changes in brain condition. In longitudinal studies of mild traumatic brain injury, the ALPS index changed from the acute to chronic phases, and its improvement was associated with recovery of sleep-related measures. In combination with enlarged perivascular spaces (ePVS), the ALPS index also improved prediction of chronic memory impairment [23]. Similarly, in normal pressure hydrocephalus (NPH), ALPS index values were lower than those in healthy controls and increased following tap testing or shunt surgery [74, 89]. These changes paralleled improvements in gait and cognitive function, suggesting that the ALPS index may respond to reversible alterations in the brain environment.

**Fig. 8:**
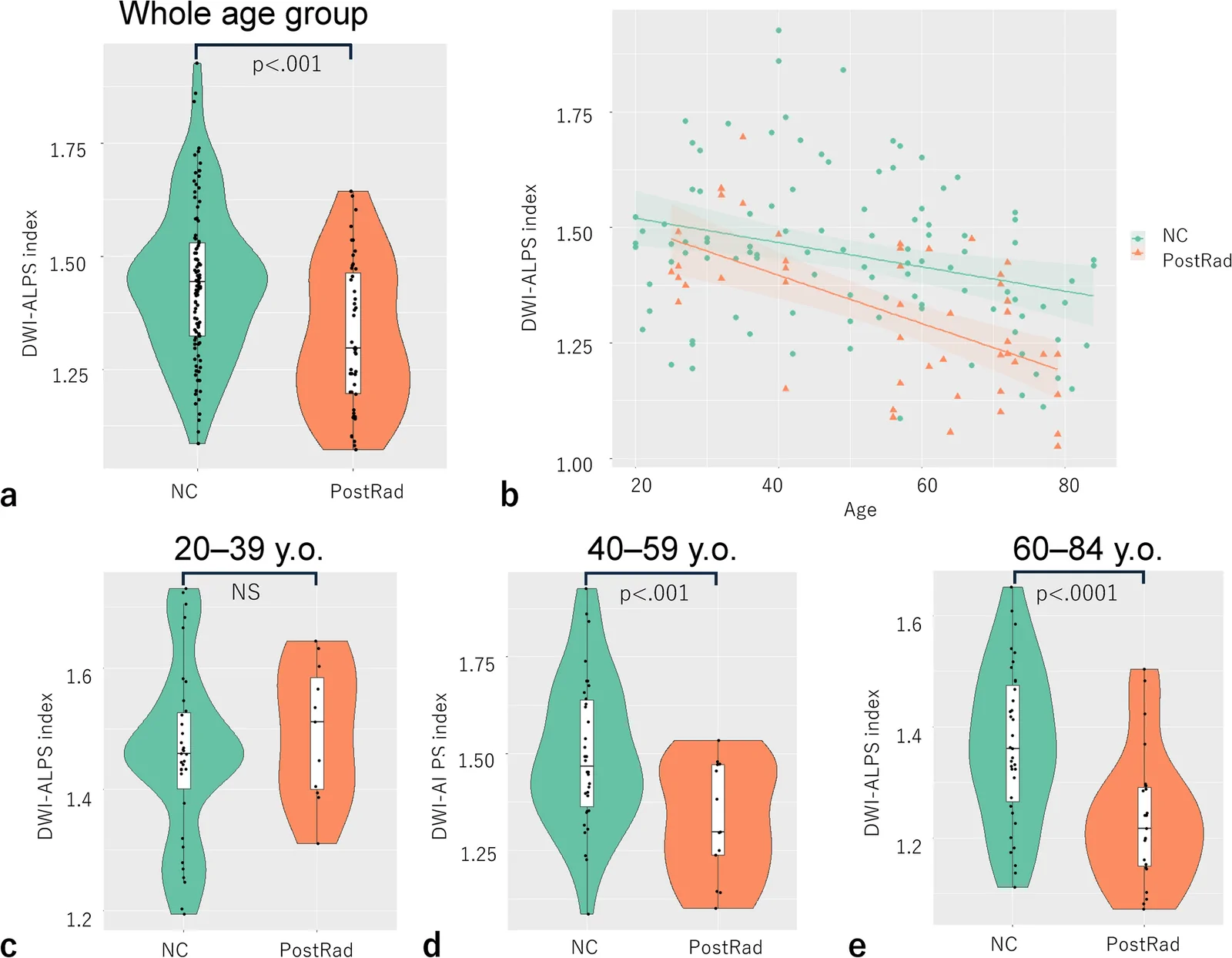
Reduction of the ALPS index after whole-brain radiotherapy and its interaction with aging. (**a**) Comparison of DWI-ALPS index values across all age groups. The post–whole-brain radiotherapy group (PostRad) showed significantly lower ALPS index values than the normal control group (NC) (*p* < 0.001). (**b**) Relationship between age and DWI-ALPS index. Both the normal control and post-radiotherapy groups demonstrated age-related decreases in the ALPS index; however, analysis of covariance revealed a significant interaction between group and age (*p* = 0.037). These findings suggest that the ALPS index may reflect not only normal aging, but also age-related tissue vulnerability and pathological alterations. (**c–e**) Age-stratified analyses. No significant difference was observed in the 20–39-year group, whereas significant reductions in the ALPS index were observed in the post-radiotherapy group within the 40–59-year and 60–84-year groups. These findings suggest that the ALPS index may demonstrate age-dependent sensitivity to pathological white matter environmental changes [91]

In addition, the ALPS index appears sensitive to neurodegeneration and brain tissue injury. In studies combining DTI-ALPS analysis with tau PET in mild cognitive impairment and the Alzheimer’s disease spectrum, lower ALPS index values were associated with tau accumulation and cognitive decline, and mediation effects involving tau pathology were also reported [28]. In patients treated with radiotherapy for nasopharyngeal carcinoma, reduced ALPS index values were associated with radiation-induced brain injury and radiation dose [90]. We also reported reductions in the ALPS index following whole-brain radiotherapy using DWI-ALPS analysis [91] (Fig. 8). Notably, these reductions were more pronounced in middle-aged and older individuals, whereas younger subjects showed no clear differences. The ALPS index also showed age-related decline and associations with white matter hyperintensities. Together, these findings suggest that the ALPS index is sensitive not only to focal radiation injury, but also to broader alterations in the brain tissue environment, including aging-related and white matter microstructural changes.

Taken together, the ALPS index demonstrates broad sensitivity to diverse pathological conditions, systemic status, aging-related changes, and longitudinal alterations over time. At the same time, these changes cannot be reduced to a single disease or pathological mechanism. Although this lack of specificity may represent a interpretive limitation, it may also indicate that the ALPS index functions as an adjunctive imaging marker relevant to brain health assessment, reflecting the integrated baseline state of the brain.

## Clinical implications

It is important to understand the ALPS index as a “fixed-point diffusion biomarker” that enables relatively stable evaluation of directional diffusivity under standardized geometric conditions. This characteristic makes the ALPS index suitable not only for cross-sectional comparisons between subjects, but also for longitudinal and large-scale population studies.

At the same time, interpretation of the ALPS index is influenced by multiple factors, including white matter microstructure, vascular geometry, and interstitial environment, making it difficult to interpret the metric in isolation. Accordingly, the ALPS index is best utilized as part of a multimodal neurofluid imaging framework in combination with modalities such as arterial spin labeling (ASL), PET, free water imaging, perivascular space assessment, and CSF or blood biomarkers [19, 28, 75, 88, 92].

Given these characteristics, the ALPS index may be better regarded not as a stand-alone diagnostic marker for a specific disease, but as an adjunctive biomarker that reflects changes in the brain tissue environment in a comparable manner. Table 2 summarizes practical considerations for DTI-ALPS studies based on current evidence (Table 2). In particular, accumulating longitudinal and intervention-related studies in conditions such as traumatic brain injury and normal pressure hydrocephalus suggest that the ALPS index may prove useful for evaluation of brain health and monitoring of brain status over time [23, 74, 89].

**Table 2:** Practical considerations for study design, acquisition, analysis, and reporting in DTI-ALPS studies

Domain	Category	Recommendation / Consideration	Rationale
Study design	Purpose of use	Define whether DTI-ALPS is used for cross-sectional comparison, longitudinal monitoring, intervention assessment, or multimodal characterization of brain tissue environment.	The biological interpretation of the ALPS index depends on study purpose and should not be reduced to a single mechanism.
	Multimodal evaluation	Consider combining DTI-ALPS with ASL, free water imaging, PET, PVS assessment, or CSF/blood biomarkers.	May improve biological interpretation.
	Longitudinal assessment	When possible, consider longitudinal or within-subject study designs rather than relying only on single time-point comparisons.	The spatially fixed-point nature of DTI-ALPS may be particularly useful for monitoring temporal changes in brain tissue environment and brain health-related conditions.
Image acquisition	Head position	Maintain consistent head positioning across subjects and sessions.	The ALPS index may change with head positioning.
	Imaging plane	Use a standardized AC–PC-based axial plane.	Imaging planes may affect reproducibility and tensor orientation.
	Phase encoding / distortion correction	Acquire reverse phase-encoding images, such as AP/PA b0 images, and apply susceptibility distortion correction such as TopUp when possible.	Reduces geometric distortion affecting ROI placement and tensor orientation.
	Diffusion acquisition parameters	Report field strength/ scanner platform, b-value, number of diffusion directions, voxel size, TE/TR, and diffusion encoding scheme.	These parameters influence directional diffusivity and ALPS index values and are essential for interstudy comparability.
Image processing and analysis	ROI placement	Prefer template-based or standardized ROI placement methods.	Reduces operator-dependent variability.
	Preprocessing pipeline	Clearly describe motion correction, eddy-current correction, and distortion correction procedures.	Preprocessing differences may affect directional diffusivity.
	Laterality	Clearly report whether the ALPS index was calculated from the left hemisphere, right hemisphere, bilateral average, or hemisphere-specific analysis.	Laterality affects interpretation and facilitates reproducibility across studies.
	Directional diffusivity analysis	Report numerator and denominator diffusivity components separately, when available.	Similar ALPS reductions may arise from different diffusivity patterns.
Interpretation and reporting	Interpretation	Interpret the ALPS index as a composite diffusion biomarker rather than a direct measure of glymphatic clearance.	The ALPS index is influenced by white matter microstructure, extracellular geometry, and perivascular-related diffusion components.
	Pattern-based interpretation	Interpret ALPS index changes according to numerator-driven, denominator-driven, or mixed/complex patterns when component diffusivities are available.	Pattern-based interpretation may help avoid attributing all ALPS index reductions to impaired perivascular or glymphatic function.
	Terminology	Use cautious terminology and avoid expressions implying direct demonstration of glymphatic dysfunction.	Encourages cautious interpretation and prevents overstatement of the ALPS index as a direct measure of glymphatic clearance.
		**Not recommended**: “glymphatic dysfunction was demonstrated”; “DTI-ALPS directly measures glymphatic clearance”; “reduced ALPS index proves impaired glymphatic function.” **Recommended**: “reduced ALPS index suggesting altered directional diffusivity related to perivascular and white matter microstructural environments”; “ALPS index reduction may reflect altered brain tissue environment or neurofluid-related tissue conditions.”	
	Reporting standards	Clearly report acquisition parameters, preprocessing pipeline, ROI methodology, laterality, and terminology used for interpretation.	Essential for transparent interpretation, reproducibility, and interstudy comparability.

## Limitations and unresolved issues

Although clinical interest in the DTI-ALPS method has increased rapidly in recent years, several unresolved methodological and biological issues remain.

First, DTI-ALPS measurements are sensitive to geometric and technical factors, including head position, imaging plane, tensor orientation, ROI placement, preprocessing pipelines, and scanner characteristics [37, 77]. Standardization of acquisition and analysis procedures—including harmonized protocols, template-based ROI placement, automated pipelines, tensor reorientation, and multicenter harmonization approaches such as ComBat—will therefore be important for future clinical applications [76, 78–80].

Second, the biological specificity of the ALPS index remains incompletely understood. The ALPS index is influenced by multiple factors, including white matter microstructure, extracellular geometry, vascular environment, and perivascular-related diffusion components, and similar ALPS alterations may arise from different pathological mechanisms [38, 52, 56]. Current diffusion MRI techniques also cannot reliably distinguish periarterial from perivenous spaces or separate perivascular diffusion from parenchymal and extracellular diffusion components. In addition, relationships between DTI-ALPS and other modalities—including intrathecal GBCA studies, ASL, free water imaging, and PET—remain incompletely clarified [19, 28, 32, 93].

Finally, although ALPS alterations have been associated with aging, cognition, sleep, neurodegeneration, and systemic biomarkers [28, 62, 68, 84], the causal mechanisms underlying these associations remain uncertain. Further studies will be required to clarify how fluid transport, white matter microstructure, vascular function, and extracellular architecture collectively contribute to the ALPS signal.

## Conclusion

The DTI-ALPS method was originally developed to evaluate directional diffusivity related to the perivascular space; however, its interpretation cannot be reduced to a single physiological process. Rather than serving as a direct measure of glymphatic function, the ALPS index should be understood as a structure-dependent composite diffusion biomarker influenced by white matter microstructure, extracellular geometry, vascular environment, and perivascular-related diffusion components.

Recent debates also suggest that glymphatic function should not be reduced to a single macroscopic bulk-flow or clearance process [94–97]. In parallel, evidence indicates that brain interstitial fluid dynamics themselves may depend on white matter architecture and extracellular geometry [9, 71, 72]. From this perspective, the structure dependence observed in DTI-ALPS may reflect physiologically relevant interactions between white matter organization and interstitial fluid dynamics rather than simple measurement bias.

In addition, the ALPS index may be conceptualized as a “spatially fixed-point diffusion biomarker” that evaluates directional diffusivity within a geometrically defined region. This characteristic supports its potential utility in longitudinal studies, brain health assessment, and multimodal neurofluid imaging. Thus, the main contribution of this review is to reposition DTI-ALPS beyond the conventional concept of glymphatic imaging and to provide a more practical and biologically nuanced framework in which the ALPS index is viewed as an integrated biomarker reflecting the broader brain tissue environment.
